# Temporomandibular disorder severity and its association with psychosocial and sociodemographic factors in Turkish adults

**DOI:** 10.1186/s12903-023-02737-1

**Published:** 2023-01-21

**Authors:** Mehmet Melih Omezli, Damla Torul, Ceren Varer Akpinar

**Affiliations:** 1 Department of Oral and Maxillofacial Surgery, Faculty of Dentistry, Ordu University, 52200 Ordu, Turkey; 2 Department of Public Health, Faculty of Medicine, Giresun University, 28000 Giresun, Turkey

**Keywords:** Temporomandibular disorders, Depression, Anxiety, Distress

## Abstract

**Background:**

There is a lack of awareness regarding temporomandibular disorder (TMD) and its association with psychological and sociodemographic factors in the Turkish population. This study aimed to evaluate the relationship between signs/symptoms of anxiety-depression, sociodemographic factors, parafunctional habits, bruxism, and the presence and severity of the symptoms of TMD in Turkish adults.

**Methods:**

The participants completed an online questionnaire consisting of sociodemographic questions, the Fonseca Anamnestic Index, and the Patient Health Questionnaire-4.

**Results:**

The mean age of the 2580 participants was 35.29 ± 12.70 years, and 63.3% were women. The frequency of the participants who showed symptoms of TMD was 69.8%. The severity of TMD symptoms was significantly greater in participants who had signs/symptoms of anxiety and depression (p < 0.05). Sociodemographic and psychological data showed an association between the presence and severity of the symptoms of TMD and sex (OR 1.52, 95% confidence interval (CI) 1.26–1.85), parafunctional habits (OR 2.64, 95% CI 2.36–2.99), bruxism (OR 3.14, 95% CI 1.78–4.90), signs/symptoms of anxiety (OR 2.30, 95% CI 1.76–3.00), and signs/symptoms of depression (OR 1.90, 95% CI 1.48–2.42).

**Conclusions:**

The results of the present study suggest that females and those who report bruxism, parafunctional habits, and signs/symptoms of anxiety-depression are more likely to show symptoms of TMD with different severity.

## Background

Temporomandibular disorder (TMD) encompasses a group of disorders affecting the temporomandibular joint (TMJ), chewing muscles, and surrounding structures [1]. The clinical manifestations of TMD, including pain, limitations in the ability to open the mouth, deviation/deflection in the path of opening, and joint sounds, can impact the quality of life negatively [2–4]. A recent meta-analysis reported the prevalence of TMD to be approximately 31% for adults [5]. The peak occurrence of TMD is seen between ages 20 and 50 years, and it predominantly affects females [2, 6].

The etiology of TMD is complex and not fully understood. Genetic, hormonal factors, stress sensitivity, and sex are reported among the factors in the multifactorial etiology [6, 7]. With the increasing recognition of the biopsychosocial model of illness, which considers the etiology of TMD within a multifactorial framework, several studies have investigated the psychosocial aspects of TMD [3, 6, 7]. The reported data indicate that psychological factors, such as depression and anxiety, play an important role in the onset, perpetuation, and prognosis of TMD [8–11]. Depression and anxiety are considered to trigger the occurrence of TMD by increasing bruxism, parafunctional habits, initiating muscular hyperactivity and joint inflammation, and changing the pain threshold by affecting the transmission of nociceptive impulses and releasing neurotransmitters [12, 13].

In addition, the symptoms of TMD, including pain, can be the cause of psychological disorders [3]. To determine the presence and severity of the symptoms of TMD, the Fonseca Anamnestic Index (FAI) is considered a reliable tool for screening in population studies [14, 15]. The FAI provides useful data in epidemiologic studies by enabling low-cost data collection that is not influenced by the researcher; it can also help to identify TMD symptoms that people are not aware of and prevent further deterioration [16]. For psychological assessment, the Patient Health Questionnaire-4 (PHQ-4) instrument, which is an ultra-short subscale of the Diagnostic Criteria for TMD (DC/TMD) Axis II, is recommended as an initial screening tool for signs/symptoms of depression and anxiety [17, 18].

There is a lack of data regarding TMD and its association with psychological and sociodemographic factors in the Turkish population. Therefore, this cross-sectional study aimed to explore the presence and severity of symptoms of TMD and its association with psychological (signs/symptoms of depression and anxiety) and sociodemographic factors, parafunctional habits, and bruxism in the Turkish adult population and to create awareness of TMD and its psychosocial aspects.

## Materials and methods

### Study participants

This cross-sectional study was approved by the Ordu University Clinical Research Ethics Committee (No. 2021/99), and informed consent was obtained from all participants. All methods were performed in accordance with the relevant guidelines and regulations of the Declaration of Helsinki. Individuals aged over 18 years who lived in provinces/districts in Turkey, had access to social media, had mastered the language in which the survey was written, and who agreed to participate in the study were included in the study. Individuals aged under 18 years and those who did not agree to participate were excluded. The sample size was calculated as 1326 by accepting the total Turkish population as 60,737,564, the prevalence as 50%, the confidence interval as 99%, and the pattern effect as 2. Accordingly, 1459 individuals were planned to be included in the sample, considering the 10% non-response margin. Individuals were reached through a questionnaire prepared via Google Forms (Google LLC, Mountain View, California, USA) using a snowball non-probability sampling method between April and June 2021. A survey was considered the most appropriate way to reach the target population, considering the COVID-19 pandemic.

### Questionnaire

The questionnaire consists of three parts: the first part focused on sociodemographic characteristics including age, sex, employment, monthly income, education level, bruxism, and parafunctional habits (pulling/keeping chin forward; squeezing mobile phone between ear and shoulder; biting tongue, cheek, or lip; talking a lot during the day; chewing gum; biting a foreign object; nail biting), the second part focused on the presence and severity of symptoms of TMD using the FAI, and the third part focused on the presence of signs/symptoms of depression/anxiety using the PHQ-4. Self-reported bruxism and parafunctional habits were answered as yes or no.

### Fonseca Anamnestic Index (FAI)

The FAI evaluates the presence or absence of symptoms caused by TMDs and their severity. The Turkish adaptation and reliability study of the scale, which was conducted in 2020, consists of 10 questions, to which the possible answers are “yes” (10 points), “sometimes” (5 points), or “no” (0 points). The total score is interpreted as follows: 0–15 points = no TMD, 20–40 points = mild TMD, 45–65 points = moderate TMD, and 70–100 points = severe TMD [19]. The questions are shown in Table 1.

**Table 1:** FAI questions

Questions			
1. Do you have difficulty opening your mouth wide?			
2. Do you have difficulty moving your jaw to the sides?			
3. Do you feel fatigue or muscle pain when you chew?			
4. Do you have frequent headaches?			
5. Do you have neck pain or stiff neck?			
6. Do you have earaches or pain in that area (temporomandibular joint)?			
7. Have you ever noticed any noise in your temporomandibular joint while chewing or opening your mouth?			
8. Do you have any habits such as clenching or grinding your teeth?			
9. Do you feel that your teeth do not come together well?			
10. Do you consider yourself a tense (nervous) person?			

### Patient Health Questionnaire-4 (PHQ-4)

The PHQ-4 was used to evaluate participants’ depression and anxiety [18]. The scale, which has been adapted into Turkish, consists of four questions: two questions assess anxiety (Over the last 2 weeks, how often have you been bothered by feeling nervous, anxious or on edge?, Over the last 2 weeks, how often have you been bothered by not being able to stop or control worrying?), and two questions assess depression (Over the last 2 weeks, how often have you been bothered by little interest or pleasure in doing things?, Over the last 2 weeks, how often have you been bothered by feeling down, depressed, or hopeless?) [20]. The PHQ-4 is a questionnaire answered on a 4-point Likert-type scale; (0) ‘not at all,’ (1) ‘several days,’ (2) ‘more than half the days,’ (3) ‘nearly every day.’ The total scores are interpreted as follows: 0–2 points = no distress, 3–5 points = mild distress, 6–8 points = moderate distress, and 9–12 points = severe distress. On each subscale, a score of 3 points or higher is considered positive for the purposes of screening for signs/symptoms of anxiety and depression.

### Data collection

Questionnaires were delivered to seven geographic regions of Turkey via e-mail and social media platforms (WhatsApp, Twitter, and Facebook).

A pilot study was conducted by administering the questionnaire to 20 individuals who were not included in the study. The pilot study was performed on a small sample from every educational level because the target population was the general population from every degree of education. It was aimed to determine the parts or medical terms that reduce the intelligibility of the questionnaire except the parts comprising the FAI and PHQ4. After that, the parts that were determined to reduce the intelligibility of the questionnaire were revised.

### Statistical analysis

The data were analyzed using version 25.0 of the Statistical Package for the Social Sciences (SPSS Inc., Chicago, IL). Descriptive statistics are given as numbers and percentages for categorical variables and mean ± standard deviation for continuous variables. Chi-square analysis was used to evaluate the relationship between the categorical variables and TMD. Multivariate regression analysis was used for advanced analysis by modeling the variables that were found to be significant. A P value < 0.05 was considered significant with 95% confidence intervals (CI).

## Results

### Study participants

Two thousand five hundred eighty individuals who completed the questionnaire were included in the study. The mean age of the participants was 35.29 ± 12.70 years, and 63.3% of the participants were women. Of the participants, 84% were university graduates, 53.3% were married, 55% were employed, and 24% were students. The monthly income of 35% of the participants was 3000 Turkish liras or less. The sociodemographic characteristics of the participants with and without TMD are shown in Table 2.

**Table 2:** Descriptive characteristics of the participants

	Total		No TMD		TMD	
	N	%	n	%	n	%
**Gender**						
Female	1633	63.3	414	53.1	1219	67.7
Male	947	36.7	365	46.9	582	32.3
**Education**						
Primary/secondary school	89	3.5	22	2.8	67	3.7
College	325	12.6	107	13.7	218	12.1
University	1578	61.2	446	57.3	1132	62.9
Master/PhD	588	22.8	204	26.2	384	21.3
**Marital status**						
Single	1206	46.7	332	42.6	874	48.5
Married	1374	53.3	447	57.4	927	51.5
**Working status**						
Retired	237	9.2	96	12.3	141	7.8
Housewife	217	8.4	50	6.4	167	9.3
Unemployed	82	3.2	33	4.2	49	32.5
Public employee	868	33.6	283	36.3	585	32.5
Student	622	24.1	150	19.3	472	26.2
Private sectore employee	554	21.5	167	21.4	387	21.5
**Monthly income (Turkish liras)**						
0–3000	908	35.2	247	31.7	661	36.7
3000–6000	720	27.9	96	12.3	190	10.5
6000–10,000	660	25.8	205	26.3	515	28.6
≥ 10,000	286	11.1	231	29.7	435	24.2
**Parafunctional habits**						
Yes	1446	56.0	332	42.6	1114	61.9
No	1134	44.0	447	57.4	687	38.1
**Bruxism**						
Yes	928	36.0	85	10.9	843	46.8
No	1652	64.0	694	89.1	958	53.2
**Depression**						
Yes	1029	39.9	174	22.3	855	47.5
No	1551	60.1	605	77.7	946	52.5
**Anxiety**						
Yes	844	32.7	118	15.1	726	40.3
No	1736	67.3	661	84.9	1075	59.7
**Depression + anxiety**						
Yes	668	25.9	81	10.4	587	32.6
No	1912	74.1	698	89.6	1214	67.4
**Age**	Mean	Std. deviation	Mean	Std. deviation	Mean	Std. deviation
	35.29	12.70	37.46	13.72	34.36	12.12

### Bruxism

**Fig. 1:**
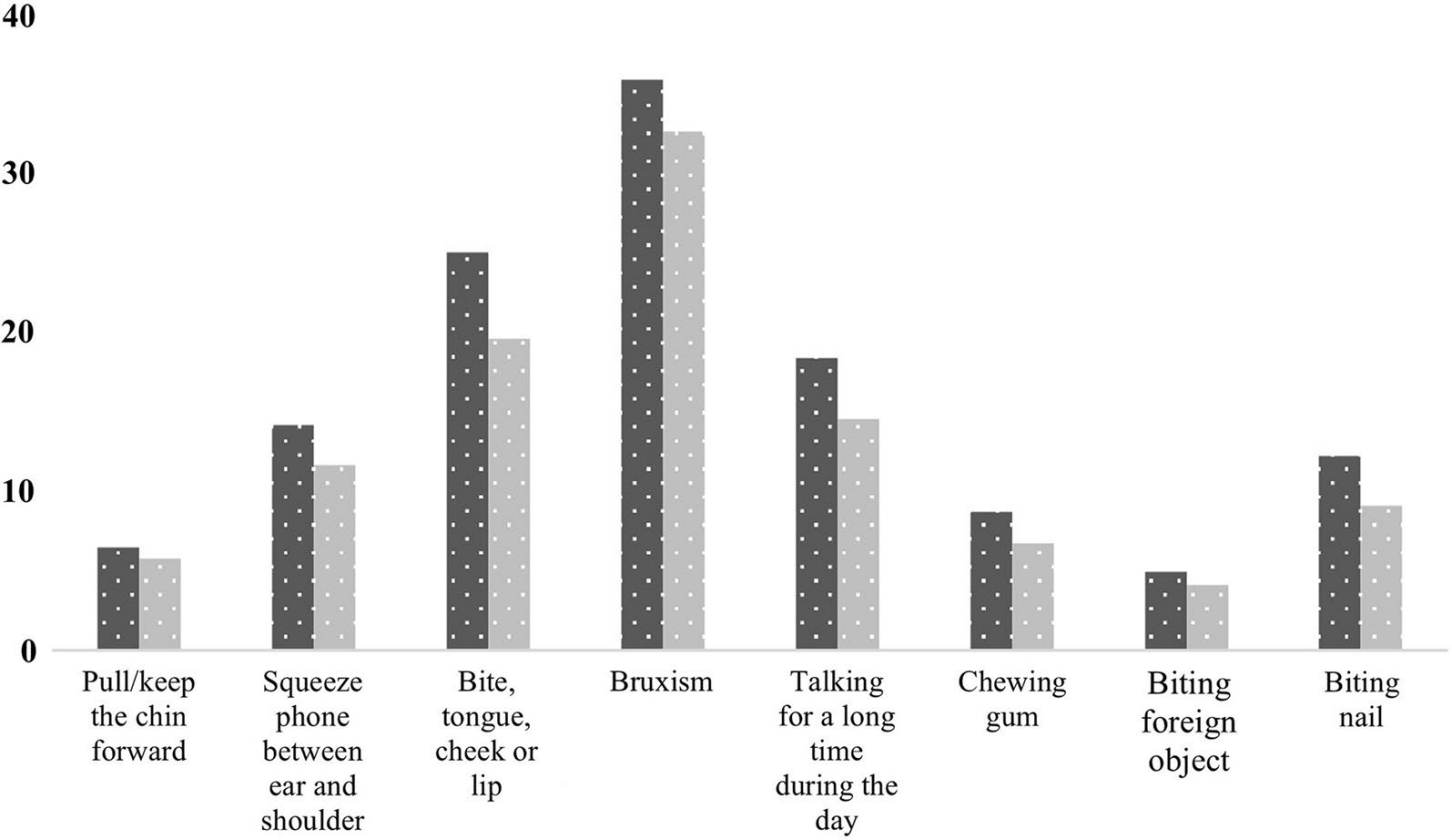
Prevalence of bruxism and parafunctional habits among participants

A total of 36% of the participants reported bruxism. Nearly half (46.8%) of the participants who reported symptoms of TMD, also reported bruxism; 10.9% of the participants who did not report symptoms related to TMD reported bruxism (Fig. 1).

### Parafunctional habits

The parafunctional habit frequencies of the participants are shown in Fig. 1; 56% of the participants stated they had at least one parafunctional habit as follows: 6.5% (n = 167) of the participants indicated that they habitually pulled/kept their chins forward; 14.2% (n = 366) of the participants reported that they habitually squeezed their mobile phone between their ears and shoulders; 25% (n = 646) indicated that they bit their tongue, cheek, or lip; 36% (n = 928) reported grinding their teeth while sleeping or awake; 18.4% (n = 474) reported talking a lot during the day; 8.8% (n = 167) reported chewing gum; 5% (n = 128) reported biting foreign objects; 12.3% (n = 317) reported biting their nails.

#### Association between psychological factors and TMD

**Fig. 2:**
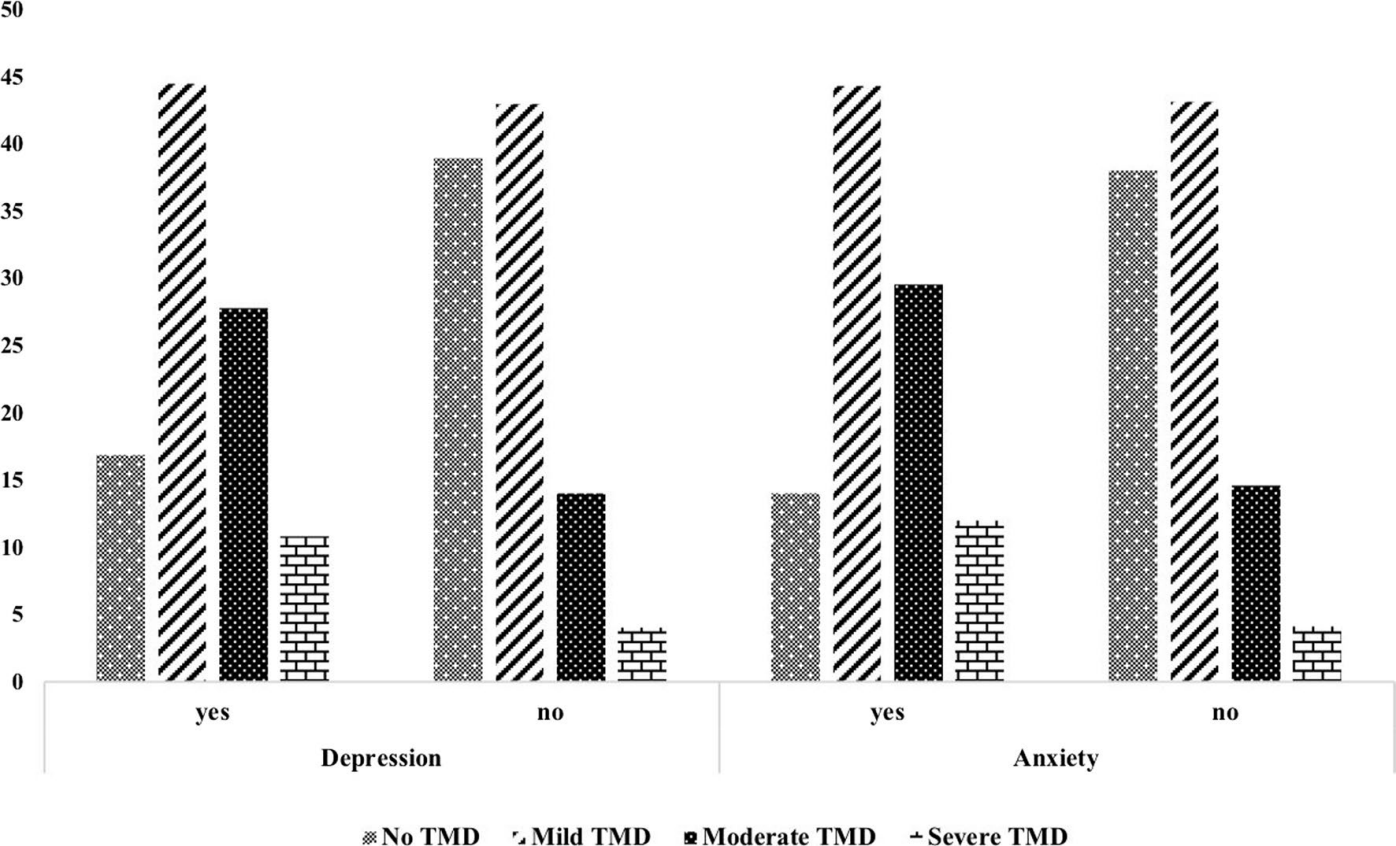
Prevalence of anxiety and depression in the participants without and with symptoms of different severities TMD

Of the participants, 69.8% reported symptoms of TMD as follows: 43.6% reported symptoms of mild TMD, 19.5% reported symptoms of moderate TMD, and 6.7% reported symptoms of severe TMD. According to the PHQ-4 scores, 75.6% of the participants had signs/symptoms of distress (anxiety/depression), including 41.9% with mild distress, 19.1% with moderate distress, and 14.6% with severe distress. According to the PHQ-4 scores, 32.7% of the participants showed signs/symptoms of anxiety, and 39.9% showed signs/symptoms of depression. Figure 2 shows the distribution of the frequency of anxiety and depression among the participants according to the presence and severity of symptoms of TMD. The presence of mild, moderate, and severe TMD symptoms was significantly higher in those with signs/symptoms of anxiety than in those without anxiety (p < 0.05). A similar trend was observed for signs/symptoms of depression (p < 0.05). The results of univariate and multivariate logistic regression analyses for TMD are shown in Table 3. The frequency of TMD symptoms was associated with sex, age, marital status, professional status, monthly income, bruxism, parafunctional habits, anxiety, and depression (p < 0.05). When multivariate analyses were performed on these variables, female sex, bruxism, parafunctional habits, and the presence of signs/symptoms of depression and anxiety were found to increase the risk for TMD symptoms.

**Table 3:** Univariate and multivariate logistic regression models of risk factors for temporomandibular disorders (N = 2580)

	N	n (%)	Crude OR	95% Cl		P	Adjusted OR	95% Cl		p
**Age**										
18–45	2005	1441 (71.9)	1.52	1.25	1.85	** < 0.001**	1.08	0.86	1.36	0.38
46–64	545	351 (64.4)	0.73	0.59	0.89	**0.02**				
65–80 (ref)	30	19 (30.0)								
**Gender**										
Male (ref)	947	582 (61.5)								
Female	1633	1219 (74.6)	1.84	1.55	2.19	** < 0.001**	**1.52**	**1.26**	**1.85**	** < 0.001**
**Education**										
Primary/secondary school	89	67 (75.3)	1.33	0.81	2.16	0.25				
College (ref)	325	218 (67.1)								
University	2166	1516 (70.0)	1.05	0.84	1.32	0.64				
**Marital status**										
Single	1206	874 (72.5)	0.78	0.66	0.93	**0.006**	0.79	0.75	1.01	0.07
Married (ref)	1374	927 (67.5)								
**Working status**										
Unemployed (ref)	536	357 (66.6)								
Student	622	472 (75.9)	1.48	1.21	1.83	** < 0.001**	1.12	0.80	1.56	0.48
Employee	1422	972 (68.4)	0.85	0.72	1.01	0.75				
**Income**										
0–3000	908	661 (72.8)	1.24	1.04	1.49	**0.01**	0.89	0.69	1.15	0.40
3000–6000	286	190 (66.4)	0.83	0.64	1.09	0.18				
6000–10,000	720	515 (71.5)	1.12	0.92	1.35	0.23				
≥ 10,000 (ref)	666	435 (65.3)								
**Parafunctional habit**										
Yes	1446	1114 (77.0)	0.45	0.38	0.54	** < 0.001**	**2.64**	**2.36**	**2.99**	** < 0.001**
No (ref)	1134	687 (60.6)								
**Bruxism**										
Yes	928	843 (90.8)	0.13	0.10	0.17	** < 0.001**	**3.14**	**1.78**	**4.90**	** < 0.001**
No (ref)	1652	958 (58.0)								
**Anxiety**										
Yes	844	726 (86.0)	3.78	3.04	4.70	** < 0.001**	**2.30**	**1.76**	**3.00**	** < 0.001**
No (ref)	1736	1075 (61.9)								
**Depression**										
Yes	1029	855 (83.1)	3.14	2.59	3.80	** < 0.001**	**1.90**	**1.48**	**2.42**	** < 0.001**
No (ref)	1551	946 (61.0)								

*ref* reference category, Bold characters: significant risk factors in univariate and multivariate analysis

The risk of showing symptoms of TMD for females was 1.52 (95% CI 1.26–1.85) fold greater than for males. The risk of symptoms of TMD for the participants who reported parafunctional habits was 2.64 (95% CI 2.36–2.99) fold greater than for those who did not report parafunctional habits. The risk of symptoms of TMD for the participants who reported bruxism was 3.14 (95% CI 1.78–4.90) fold greater than for those who did not report bruxism. The risk of symptoms of TMD increased 2.30 fold (95% CI 1.76–3.00) in the presence of signs/symptoms of anxiety and 1.90 (95% CI 1.48–2.42) fold in the presence of signs/symptoms of depression (Table 3).

## Discussion

This cross-sectional study explored the presence and severity of symptoms of TMD and the association between TMD and psychological (signs/symptoms of anxiety-depression) and sociodemographic factors, parafunctional habits, and bruxism in the Turkish adult population. The results showed that 70% of the participants had mild-to-severe TMD, and females and those who reported bruxism, parafunctional habits, or signs/symptoms of anxiety-depression were more likely to develop TMD. The prevalence of symptoms of TMD in this study was significantly higher than indicated by the results of studies of different populations [21–24] and by the results of a previous study of the Turkish population [25]. This difference may originate from the differences in the samples, the methodology, and the evaluation methods used in the studies. However, this result may also be explained by the COVID-19 pandemic, which has had a negative impact on people’s mental health [26, 27].

### Psychological factors and TMD

The role of psychological factors in TMD has not been entirely elucidated. Several studies have demonstrated an association between psychological factors and TMD, with higher depression/anxiety levels found in patients with TMD [3, 8, 13, 28]. On the other hand, other studies have failed to find any such association [29, 30]. In this study, a significant association was found between the presence and severity of symptoms of TMD and signs/symptoms of depression/anxiety. The prevalence of the signs/symptoms of depression and anxiety among the participants who had TMD symptoms in different severity were 47% and 40%, respectively. This result is similar to previous studies that reported the prevalence of depression in patients with TMD as 21–60% [31] and the prevalence of anxiety as 12–75% [3, 17, 32]. In addition, the comorbidity of anxiety and depression was reported to increase the prevalence and severity of TMD compared with the presence of either anxiety or depression alone [17, 33]. The results of the present study confirmed this finding.

In the current study, signs/symptoms of anxiety and depression were found to be associated with the presence and severity of symptoms of TMD, with a higher risk of 2.30 and 1.90 fold, respectively. This result is in line with the findings of previous studies with different designs that investigated the association of depression and anxiety with TMD [8, 9, 11, 13, 34]. Although further longitudinal research is needed to determine the causal relationship between physiological factors and TMD, evidence from previous studies and the present study reinforces the importance of understanding psychological factors in the management of patients with TMD.

### Age/sex and TMD

The relationship between TMD and age/sex is well-established in the literature. Several studies have found that young females reported TMD symptoms more frequently than others. In a cross-sectional study conducted on the Brazilian population, the majority of individuals with TMD were found to be female (66%) and young adults (85%) [23]. In another study on Canadian subjects, females and those in younger age groups were more likely to report one or more TMD symptoms [24]. In a study on the Finnish population, female sex was found to be significantly associated with the occurrence of nearly all the signs of TMD [22]. However, in another cross-sectional study on the Turkish population, age-related differences in TMD symptoms were not observed, and sex differences were observed only for the symptom of pain [25]. Similarly, in their study on the Hong Kong Chinese population, Pow et al. [35] found no difference in TMD symptoms between the sexes. However, in our study, young individuals (80%) and females (67%) constituted the majority of the individuals with had symptoms of TMD. In a meta-analysis, Bueno et al. [36] reported an increase of 2.2-fold in the risk of TMD in females compared with males. Resende et al. [3] found a 3.5-fold higher OR for the incidence of TMDs in females compared with males. In a cross-sectional study, Goncalves et al. [4] observed that the risk of females reporting at least one and up to three or more TMD symptoms was 1.31–2.49 fold higher, respectively, than for males. The risk of having symptoms of TMD was found 1.52 fold higher in females than in males in this study. In the literature, the predominance of TMD in females has been attributed to genetic, hormonal, psychosocial, and cultural factors, as well as differences in pain perception/thresholds and treatment-seeking behaviors between the two genders; these factors are also likely at play in the present study [1, 12, 36]. Regarding the effect of age, in the current study, the significant effect found with the univariate analysis was not observed in the multivariate analysis. This finding may indicate that when factors such as depression, anxiety, and sex associated with TMD are controlled, the risk associated with young age disappears.

### Parafunctional habits, bruxism and TMD

In the present study, parafunctional habits and bruxism were found in nearly 62% and 47% of the individuals who reported mild-to-severe symptoms of TMD. Parafunctional habits and bruxism were found to increase the risk of present symptoms of TMD by 2.64 and 3.14 fold, similar to the results of previous studies [37–39]. This increased risk is possibly associated with greater and more severe signs/symptoms of anxiety and depression in individuals who present with symptoms of TMD, which may trigger parafunctional activities and bruxism. Similarly, Kmeid et al. [21] suggested that the presence and severity of TMD were associated with greater distress, bruxism, and the number of hours spent on the phone per day in the Lebanese population.

### Strengths and limitations

The strength of this study is that it evaluated factors that might be associated with TMD by controlling for these factors through a regression model. Thus, the authors provide reliable results by eliminating the effects of confounding factors. The findings of the study should be interpreted with caution because of certain limitations. First, the study was conducted during the most intense period of the COVID-19 pandemic. For this reason, online data collection was used instead of a face-to-face method. Thus, a selection bias is probable because the sample is not representative of the entire population; the online data collection method may have limited the participation of certain disadvantaged groups, such as those without internet access and those with low socioeconomic status. Second, the study data are cross-sectional; in the future, longitudinal study designs are needed to confirm the causal relationship between the study variables and TMD. Third, because we aimed to perform a general screening on the adult population, individuals with a recent trauma to the face, underlying motor, autoimmune, and neurologic conditions that could influence the occurrence of the symptoms of TMD and thereby the results of the study were not excluded. Although the PHQ-4 used in this study is a reliable screening tool for depression and anxiety symptoms/signs, its reliability as a diagnostic tool is limited. Finally, information bias might be present because the assessment is based on information provided by the participants.

## Conclusion

This study considers self-reported TMD symptoms and psychological symptoms as roughly screened, no diagnoses of either TMD, bruxism, anxiety, or depression were made. Despite its limitations, this study showed an association between symptoms of TMD and sex, bruxism, parafunctional habits, signs/symptoms of anxiety, and depression. The results showed that females and those who report bruxism, parafunctional habits, and signs/symptoms of anxiety-depression were more likely to develop TMD symptoms. This result supports the biopsychosocial model and reinforces the need for a multidimensional evaluation of TMD and a multidisciplinary approach in the management of patients with TMD, as well as the need to increase awareness of the role of psychosocial factors in TMD. Also, further clinical studies are needed to determine the real physiological mechanism that links stress, anxiety-depression, TMD, and bruxism more clearly.

## Data Availability

The datasets used and/or analyzed during the current study are available from the corresponding author on reasonable request.
